# Gradient Index Metasurface Lens for Microwave Imaging

**DOI:** 10.3390/s22218319

**Published:** 2022-10-30

**Authors:** Srijan Datta, Antonello Tamburrino, Lalita Udpa

**Affiliations:** 1 Department of Electrical and Computer Engineering, Michigan State University, East Lansing, MI 48824, USA; 2 Department of Electrical and Information Engineering, Università degli Studi di Cassino e del Lazio Meridionale, 03043 Cassino, Italy

**Keywords:** metamaterials, lenses, focusing, microwave imaging, nondestructive testing

## Abstract

This paper presents the design, simulation and experimental validation of a gradient-index (GRIN) metasurface lens operating at 8 GHz for microwave imaging applications. The unit cell of the metasurface consists of an electric-LC (ELC) resonator. The effective refractive index of the metasurface is controlled by varying the capacitive gap at the center of the unit cell. This allows the design of a gradient index surface. A one-dimensional gradient index lens is designed and tested at first to describe the operational principle of such lenses. The design methodology is extended to a 2D gradient index lens for its potential application as a microwave imaging device. The metasurface lenses are designed and analyzed using full-wave finite element (FEM) solver. The proposed 2D lens has an aperture of size 119 mm (3.17λ) × 119 mm (3.17λ) and thickness of only 0.6 mm (0.016λ). Horn antenna is used as source of plane waves incident on the lens to evaluate the focusing performance. Field distributions of the theoretical designs and fabricated lenses are analyzed and are shown to be in good agreement. A microwave nondestructive evaluation (NDE) experiment is performed with the 2D prototype lens to image a machined groove in a Teflon sample placed at the focal plane of the lens.

## 1. Introduction

Lenses are devices known for their ability to focus incident electromagnetic (EM) waves and form images of an object [1]. They have been studied and widely used in the optical regime of the electromagnetic spectrum for centuries. Since the 1940s, lens designs at microwave frequencies have been investigated [2,3,4,5,6,7] and implemented in a wide range of applications such as imaging [8,9], radar systems [10,11], material characterization [12,13] and non-destructive testing [14]. Traditionally, dielectric materials have been used to engineer lenses in the microwave regimes, but they suffer from heavy and bulky profiles, and their machining processes are expensive as well [15].

As alternatives to conventional lenses, metamaterials have been researched extensively in recent years as means of controlling the propagation of EM waves ranging from microwave to optical [16]. These are engineered structures, which consist of periodic arrangement of subwavelength scatterers or unit cells, whose size is much smaller than the operating wavelength λ. Based on the effective medium theory, a periodic arrangement of subwavelength scatterers can be characterized by effective permittivity and effective permeability. The unit cell size a should be less than λ/4 for such inhomogeneous periodic materials to behave like a homogenous medium to incident waves [17,18]. The unit cells of metamaterials have also been referred to as meta-atoms because of their analogy to atoms of crystalline materials. The bulk EM properties of such metamaterials depend on the contents and arrangement of unit cells in contrast to material composition in the atomic scale such as that of dielectric materials. Hence, they can be tailored to have specialized EM characteristics including novel lensing applications, which are difficult or impossible to achieve with lenses made of conventional materials. The concept of 3D bulk metamaterials was judiciously extended to 2D metasurfaces, which consist of subwavelength unit cell placed on a surface or an interface [19]. Metasurfaces provide an attractive alternative to metamaterials as they take less physical space and exhibit lower losses.

Research in metamaterials and metasurfaces was stimulated by the theoretical work of V. Veselago et al. in the 1950s [20,21,22] and later by the realization of engineered metamaterials by Smith et al. at the turn of the century [23,24,25,26,27]. The use of metamaterials as lenses was first suggested by Pendry in [28], where he showed theoretically that a slab made of negative index metamaterials (NIM) can act as a ‘perfect lens’ for a diverging source as both the propagating and evanescent waves will contribute to the resolution of the image. Although losses associated with engineered metamaterials make it impossible to realize perfect lensing conditions [29,30], sub-diffraction imaging using NIM lenses is still achievable as shown by various studies [31,32,33,34]. However, despite their super resolution capabilities, NIM lenses still have one fundamental limitation: the inability to focus plane waves into a focal spot, which forms the basis of the Fourier transform and imaging capabilities of a conventional lens [35]. NIMs also tend to be narrowband and highly dispersive structures since they operate in the resonant region of the unit cells.

In 2005, the concept of NIM was extended to graded metamaterials, where it was shown that metamaterials whose effective electromagnetic properties vary spatially can also be fabricated by introducing a slight change in the properties of each successive unit cell [36]. This paved the way for numerous unprecedented applications of metamaterials, including GRIN metamaterial lenses, which can provide phase compensation mechanisms and bring plane waves into focus [37]. A GRIN metamaterial lens also operates in non-resonant regions of its unit cells and hence overcomes the narrowband and large transmission losses associated with NIM lenses [38].

The idea of GRIN lenses was theorized by Maxwell back in 1854, when he described remarkable imaging capabilities of a sphere with a radially symmetric refractive index [39]. Such gradual refractive index variation can be used to construct flat lenses, where bending of incident waves is achieved through the refractive index contrast rather than the curved surface of conventional lenses. Classic examples of GRIN lenses include the Fresnel lens, Wood lens and Luneburg lens [40,41,42,43]. The advent of metamaterials and metasurfaces has renewed interest in research of GRIN lenses and inspired numerous works [44,45,46,47,48,49,50]. The tailorable properties and flat geometry of metasurfaces has provided ground-breaking engineering potential for GRIN lens designs, which was not possible with conventional dielectrics.

This paper presents the design, simulation and experimental validation of GRIN metasurface lenses operating at 8 GHz for microwave imaging applications. The proposed lenses use PCB technology and thus constitute a low-cost, low-profile and lightweight design. Section 2 provides a brief summary of the underlying principles of GRIN lens operation. In Section 3, the unit cell design of the metasurface lens is introduced and the results of a parametric simulation study are reported. The unit cell simulation results were used to guide the design of a 1D followed by a 2D gradient metasurface lens with a predetermined focal length. The focusing action of the lenses are reported using full wave FEM solver Ansys HFSS. Prototype of the lenses were fabricated for experimental verification and the corresponding results are presented in Section 4. A microwave NDE experiment with the 2D metasurface lens is reported at the end to demonstrate its capability as a microwave imaging device.

## 2. Theory

**Figure 1:**
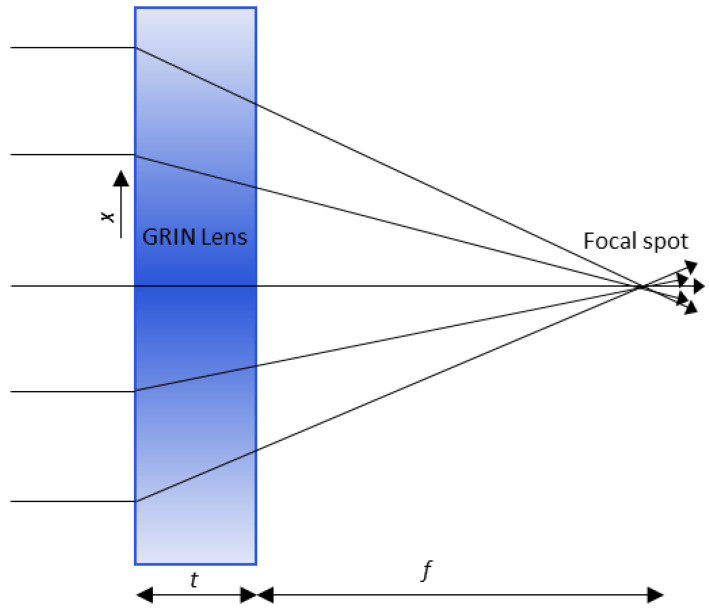
GRIN Lens operation. Shading of the lens indicates the gradient in the index: darker shading corresponds to a higher refractive index value.

A GRIN lens consists of a planar slab with spatially varying refractive index as shown in Figure 1. Consider a plane wave that consists of parallel rays incident on a GRIN lens. The optical paths of the rays are curvilinear but can be approximated to linear paths for a very thin lens. Using geometric optics, the optical path *p*(*x*) of an arbitrary ray located at *x* can be written as [47].

(1) $$p\left(x\right)=\sqrt{f^{2}+x^{2}}+n\left(x\right)t$$

where f is the focal distance and n(x) is the spatial distribution of the refractive index. For all the rays to converge to a single focal point, the optical path lengths of arbitrary off-axis rays should equal to those passing exactly through the optical axis. This results in the gradient index profile n(x) for GRIN lenses is expressed as,

(2) $$n\left(x\right)=n_{0}-\frac{1}{t}\left[\sqrt{f^{2}+x^{2}}-f\right]$$

where $n_{0}$ is the maximum refractive index at the center of the lens. EM waves can be approximated as rays using geometric optics for optical lenses as lens dimensions are many orders of magnitudes larger than wavelength of light. However, full-wave electromagnetic theory must be considered to describe lenses that operate at microwave frequencies. Wave analysis takes into account effects such as interference and diffraction which become significant when the size of the lens is comparable to the wavelength [51]. Similar to optical path analysis, the phase advance of all the rays from the incident lens surface to the focal spot should be equal in this case. For this, the spatial distribution of the phase shift, φ across the lens should obey the following equation [46].

(3) $$\varphi \left(x\right)=\frac{2\pi \left(\sqrt{f^{2}+x^{2}}-f\right)}{\lambda }$$

where x is the position on the lens in the x-direction and the center of the lens is at x=0, f is the focal length of the lens, and λ is the operating wavelength.

## 3. Design

### 3.1. Unit Cell

**Figure 2:**
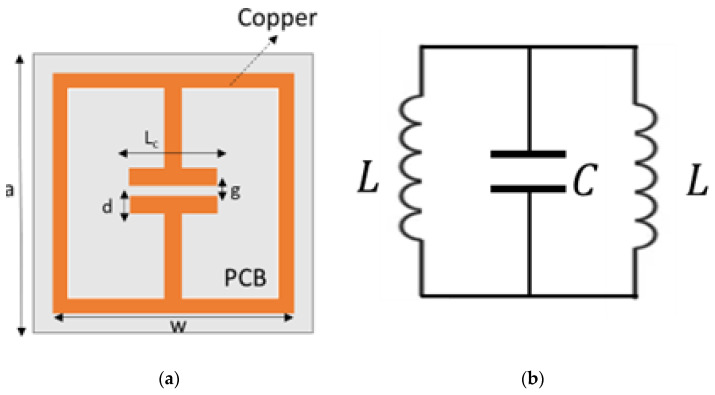
(**a**) ELC unit cell of proposed GRIN Metasurface lens (**b**) Equivalent circuit of ELC resonator unit cell.

The unit cell used for the proposed GRIN lens is an electric-field-coupled LC (ELC) resonator first proposed by Schurig et al. [52] and is shown in Figure 2a. This resonator has a split-gap at the center of the structure providing capacitance (which couples strongly to an applied electric field parallel to the split-gap) and two loops in parallel on either side to provide inductance in the structure [53]. An equivalent circuit of the resonator is shown in Figure 2b. The resultant resonant frequency $f_{o}$ can be expressed as

(4) $$f_{o}=\frac{1}{\pi \sqrt{2LC}}$$

where L/2 and C are the total inductance and capacitance of the structure, respectively. When the resonator is excited by a uniform magnetic field parallel to the plane of the structure, currents induced in the two loops are in opposite direction leading to zero magnetic moment. Thus, the structure does not provide magnetic coupling, and only the capacitive element C drives its fundamental resonance according to Equation (4). 

The split-gap dimensions g can be varied to change the $\epsilon_{reff}$ and hence the refractive index ($n^{2}=\mu_{eff}\epsilon_{eff}$) and resonating frequency $\omega_{o}$ of the unit cell [54]. A smaller split-gap will correspond to a larger capacitance and consequently a higher refractive index $\left(C\propto \epsilon_{eff}\right)$. Hence, a symmetric array of such ELCs, with increasing split-gap from the middle, will generate the required refractive index profile for focusing. Incident waves from both the edges of the lens will be refracted towards the higher refractive index unit cells at the center, thus producing a focusing action. 

**Figure 3:**
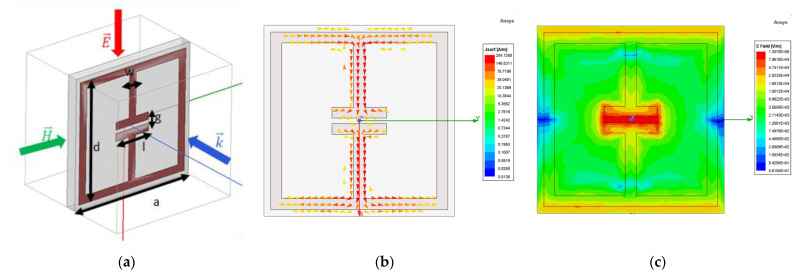
(**a**) HFSS unit cell model (**b**) Surface current density (**c**) Electric field distribution.

**Figure 4:**
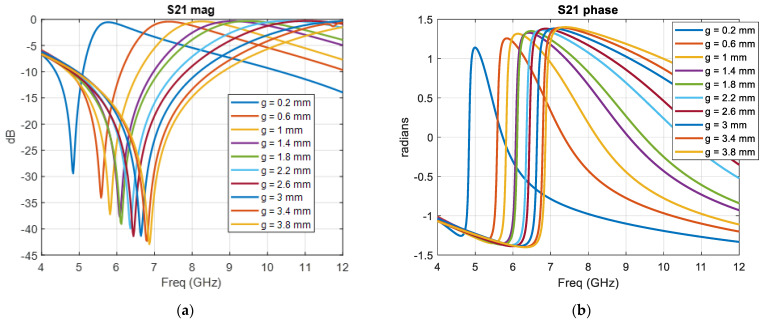
HFSS Parametric simulation results (**a**) S_21_ magnitude (**b**) S_21_ phase.

The ELC unit cell HFSS model, with the incident wave polarization and dimensional parameters, is shown in Figure 3a. The dimensional parameters for the unit cell design are: *a* = 7 mm, *d* = 6.5 mm, *l* = 2 mm. Periodic boundary conditions were used to obtain the scattering parameters for the unit cell in a metamaterial arrangement. Figure 3b,c plot the surface current density and electric field distribution, respectively, as obtained from HFSS numerical results. The surface current density plot shows that there is no net circulation of current in the unit cell due to clockwise and counterclockwise components in adjacent areas of the structure as expected. The electric field distribution plot shows a strong local field enhancement in the capacitive gap at the center of the unit cell. The frequency response of the unit cell was varied by varying the split-gap *g*. The nominal split-gap *g* for the highest refractive index unit cell was taken as 0.2 mm due to fabrication limits. The PCB material used was FR4 with a thickness of 0.6 mm and trace width (*w*) of 0.4 mm. A parametric unit cell study was performed for varying values of split-gap *g*, ranging from 0.2 mm to 4 mm to obtain varying phase shifts and determine the frequency of operation of the lens. Figure 4a,b show the parametric S_21_ magnitude and phase results for 10 representative cases obtained using HFSS. 8 GHz was chosen as the frequency of operation from the parametric results to maximize transmission through the lens (S_21_ magnitude) and achieve high phase shift between the individual unit cells (S_21_ phase) simultaneously.

### 3.2. Metasurface Lens

**Figure 5:**
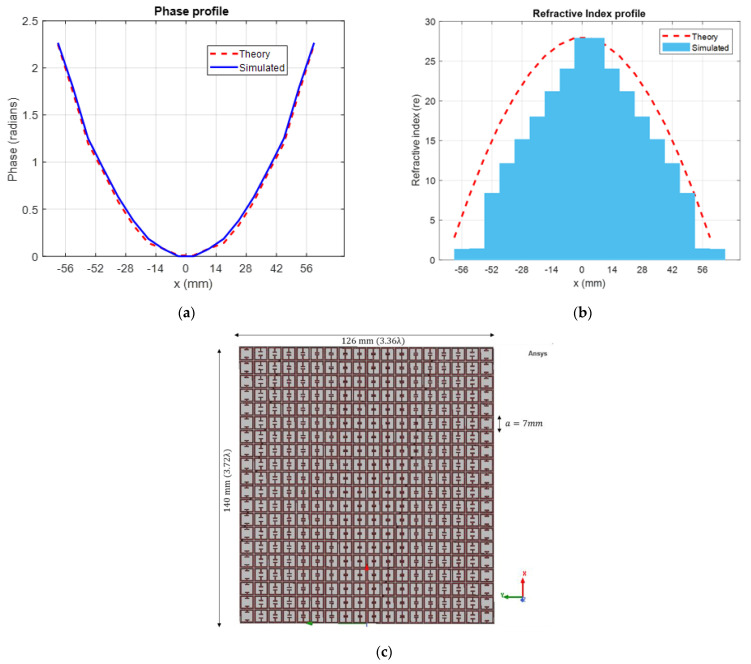
(**a**) Theoretical and simulated phase gradient (**b**) Theoretical and extracted refractive index gradient (**c**) Proposed 1D GRIN metasurface lens design.

The desired phase profile of 1D gradient index lens of thickness 0.6 mm operating at 8 GHz for a focal distance of 110 mm follows (3) and is plotted in Figure 5a. The numerical phase shift results from the model-based parametric study were used to determine unit cells with appropriate split-gaps and obtain an approximation to the desired phase profile. The proposed GRIN metasurface lens design consists of 18 × 20 unit cells in the *x-y* plane as shown in Figure 5c. The lens has a total dimension of 126 mm (3.36λ) × 140 mm (3.72λ) × 0.6 mm (0.016λ). The simulated phase profile of the final 1D GRIN lens design is also plotted in Figure 5a. A higher phase shift is observed for larger split-gap unit cells as expected.

Electromagnetic parameter retrieval according to Appendix of [34] was carried out for the 18 unit cells to extract the refractive index profile. The highest refractive index (*n_o_* in Equation (1) at the center of the GRIN lens is 27.91 for the proposed design at 8GHz. The simulated refractive index profile is plotted in Figure 5b. The theoretical refractive index profile according to (2) is plotted as well to demonstrate the correlation between geometric optics and full-wave electromagnetism. The difference in the theoretical and simulated gradient index profile is due to the approximations of EM waves as straight rays in geometric optics, which does not consider effects such as scattering and diffraction. Although it has been argued that assigning bulk material properties to metasurfaces using a variant of Nicholson Ross Weir method may produce ambiguous results [55,56], they can still be used to characterize metasurfaces if the thickness of the metasurface remains constant [19].

**Figure 6:**
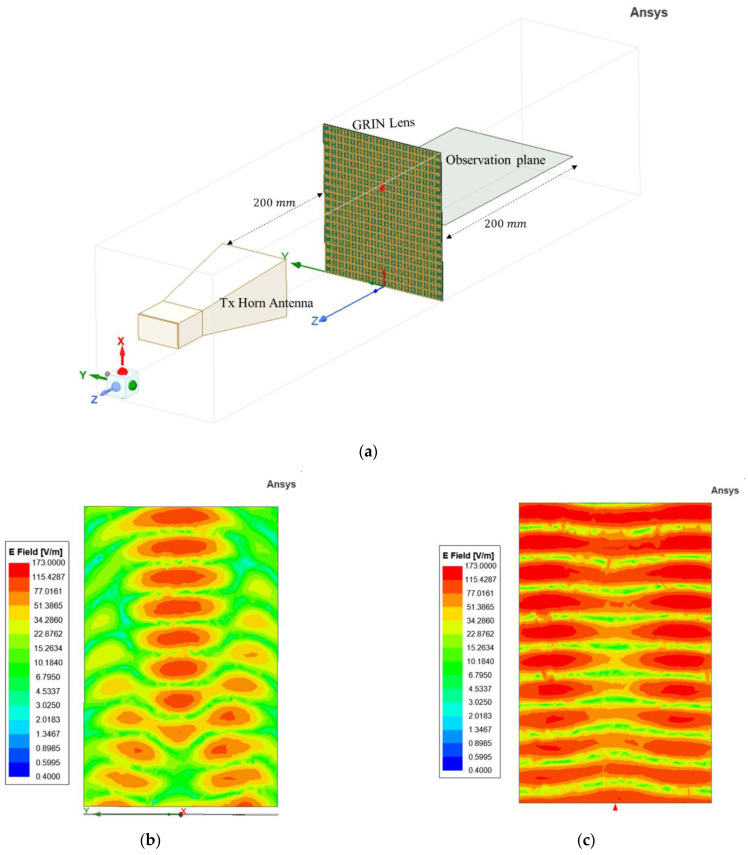
(**a**) HFSS setup for focusing simulation of 1D GRIN lens. (**b**) Simulated E field distribution with lens. (**c**) Simulated E field distribution without lens.

Full-wave simulation of plane waves incident on the lens was performed in HFSS to illustrate the focusing action. The geometrical setup modeled by HFSS is shown in Figure 6a. The GRIN lens was placed on the *x-y* plane and centered at the origin. The gradient of index is along the *y* direction, while the wave propagation is along *z* direction. A horn antenna with its *E* field polarized along the *x*-axis was used as the source of incident EM waves. The antenna was kept at a far-field distance of 200 mm (5λ) from the lens to ensure plane waves are incident on the lens surface. Open boundary conditions were used to simulate the structure in free space. The magnitude of the simulated *E* field distribution is plotted on a *y–z* observation plane on the other side of the lens. In Figure 6b, the waves can be seen to refract towards the center after passing through the GRIN lens and come to focus close to the designed focal length of 110 mm (2.93λ) from the plane of the lens. Figure 6c shows the full-wave simulation without the GRIN lens. *E* field distribution without the lens shows the incident plane waves from the horn antennas propagating without any focusing action.

**Figure 7:**
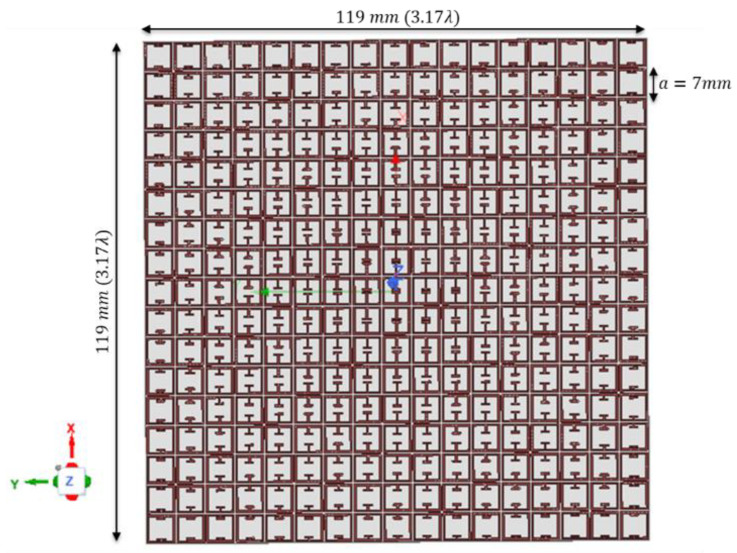
Proposed 2D GRIN Metasurface Lens for microwave imaging.

The GRIN lens described in the previous section has a gradient index in only one direction and hence the focusing action was in the azimuthal (horizontal) plane only. The same design concept was extended for a two-directional gradient metasurface lens design and is reported in this section. The focusing action for this case will occur in both the vertical and horizontal plane and hence a point focus can be obtained for microwave imaging applications. The proposed 2D GRIN lens design is shown in Figure 7 and consists of 17 × 17 unit cells with dimensions of 119 mm (3.17 λ) × 119 mm (3.17 λ) × 0.6 mm (0.016 λ).

**Figure 8:**
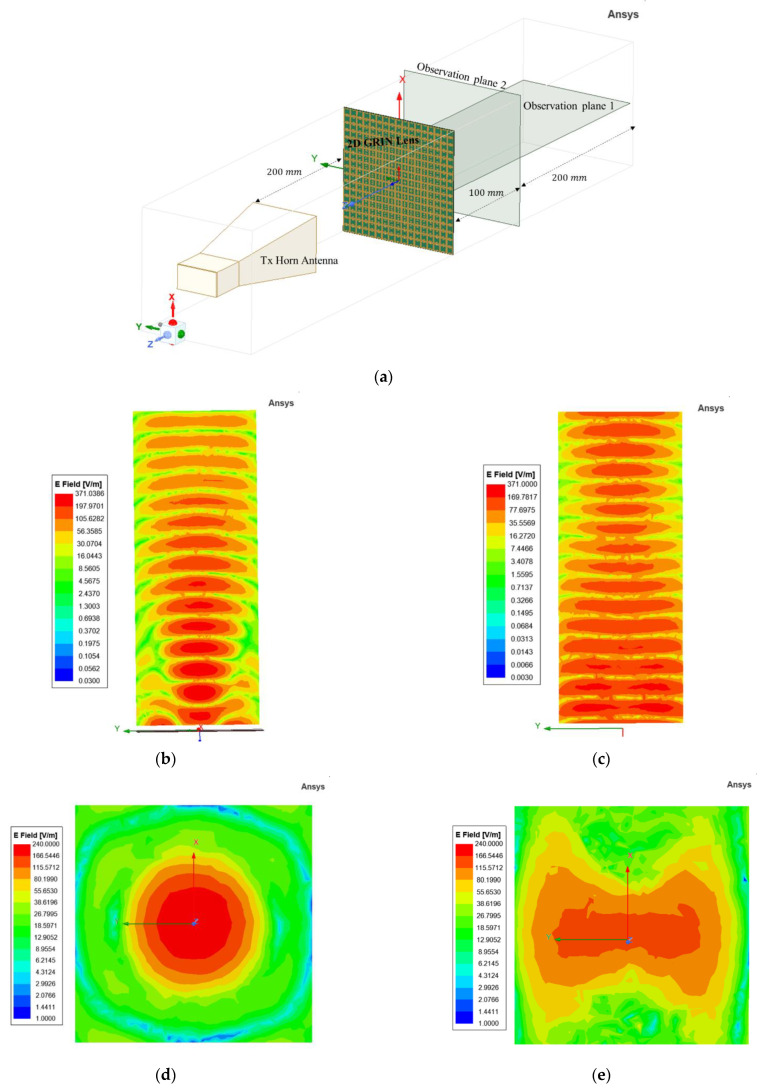
(**a**) Ansys HFSS setup for proposed 2D GRIN Metasurface Lens. (**b**) Azimuthal plane electric field distribution with lens and (**c**) without lens. (**d**) Vertical plane electric field distribution with lens and (**e**) without lens.

The HFSS model setup to validate focusing action is shown in Figure 8a. Two observation planes (both horizontal and vertical) were considered in this case. The solution frequency is 8 GHz, which is same as the 1D GRIN lens. The electric field distribution in the horizontal plane are shown in Figure 8b,c. A similar focusing action is observed with the 2D GRIN lens, while the free space results show the incident plane waves without any focusing. The predetermined focal length for this design is 100 mm. The vertical observation plane was placed at the focal plane (100 mm from the GRIN metasurface lens) and the electric field distribution are plotted in Figure 8d,e to observe the point-focusing action. Symmetric refraction of incident waves produces a circular confinement of an electric field in the focal plane as observed in Figure 8d. The electric field distribution without the 2D GRIN lens is plotted as well (Figure 8e) to indicate the resolution enhancement.

## 4. Experiment

### 4.1. Focusing 

**Figure 9:**
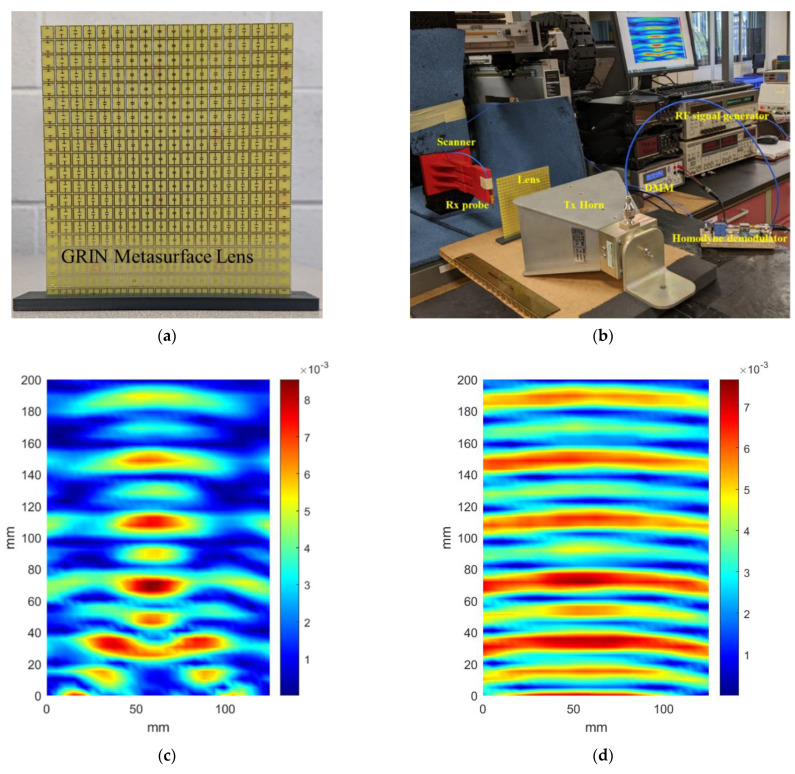

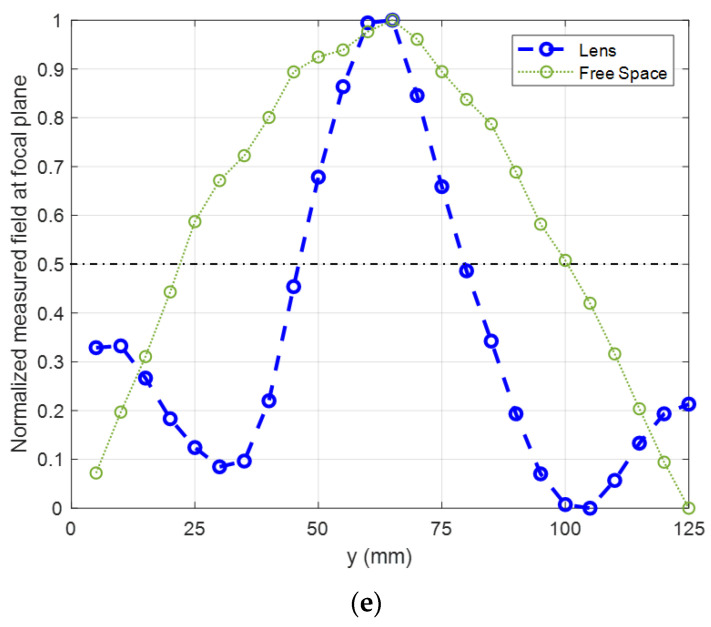
(**a**) Fabricated GRIN Metasurface Lens. (**b**) Experiment setup using homodyne imaging system. (**c**) Measured field distribution with lens. (**d**) Measured field distribution without lens. (**e**) Normalized measured field at focal plane.

A prototype of the proposed 1D GRIN metasurface lens was fabricated at first for experimental verification. The fabricated lens is shown in Figure 9a. A homodyne detection scheme described in [34] was used to perform microwave imaging experiments at 8 GHz. A wideband horn antenna was used as the source of plane waves. The horn was kept at 200 mm from the metasurface lens. A quarter wavelength monopole was used as the receiver to sample the field after it passes through the lens. The experimental setup is shown in Figure 9b. Figure 9c,d show the measured field distributions with and without the lens. From the plots, focusing action of the fabricated metasurface lens is clearly observed and they match well with the numerical results shown in Figure 6. The cross range field distribution in the focal plane (110 mm) with and without the lens is plotted in Figure 9e. The full width at half maximum (FWHM) at the focal plane with the lens is 33 mm (0.88λ) and is 77 mm (2.04λ) without the lens, indicating a resolution enhancement by a factor of 2.33 that is achieved with the 1D GRIN lens in the azimuthal plane.

**Figure 10:**
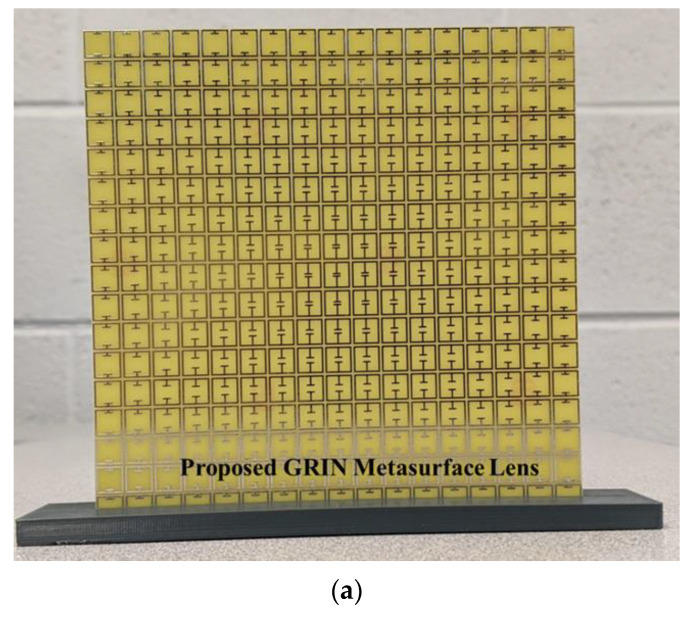

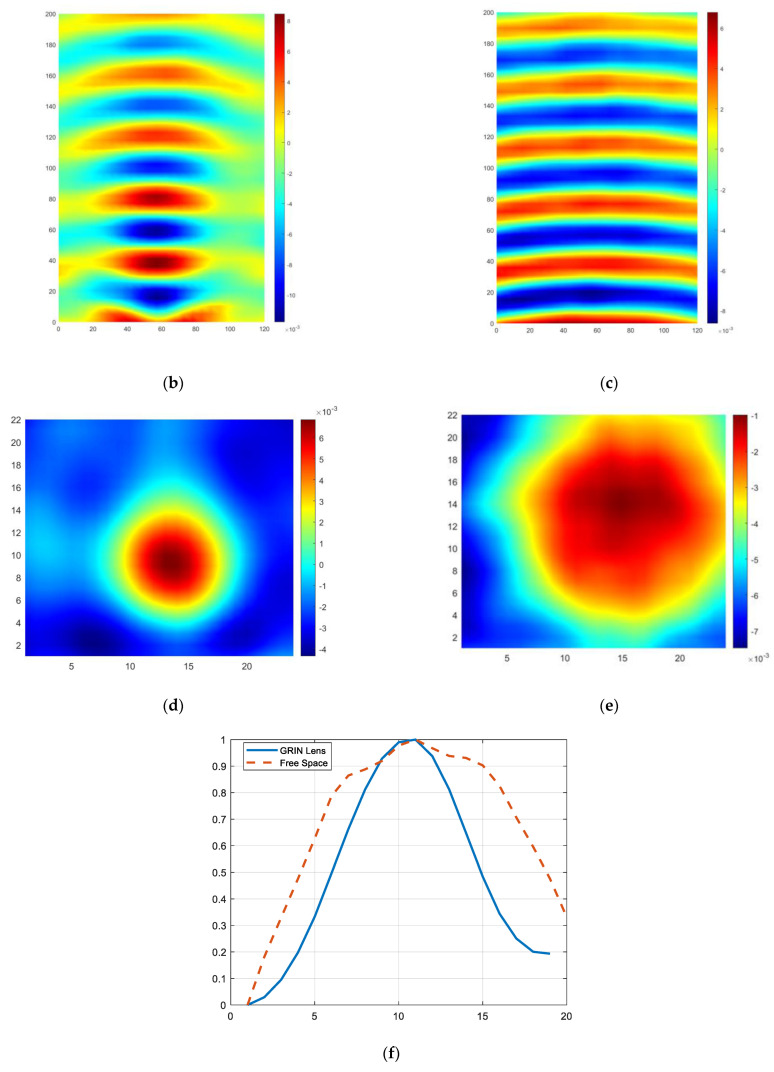
(**a**) Fabricated 2D GRIN Metasurface Lens. (**b**) Field distribution in horizontal plane with lens and (**c**) without lens. (**d**) Field distribution in vertical (focal) plane with lens and (**e**) without lens. (**f**) Cross- range intensity profile comparison of lens and free space at focal plane.

The prototype of the 2D GRIN lens is shown in Figure 10a. The homodyne setup described previously was used to perform the experiments. A horn antenna kept at a distance of 200 mm was used as the source of incident plane waves. A quarter wave monopole was used to sample the field distribution in the horizontal as well as the vertical plane. The experiments were performed without the lens as well to validate the focusing action of the lens. The horizontal field distribution is plotted in Figure 10b,c and compares well with the simulation results shown in Figure 8. The vertical observation plane was located 100 mm from the lens surface and the experimental results are shown in Figure 10d,e. A circular focal spot was obtained with the 2D GRIN lens as expected (Figure 10d). The experiment was repeated without the lens to calculate the resolution enhancement. The FWHM with lens is 40 mm (1.2λ) and without lens is 70 mm (1.86λ), thus indicating a resolution enhancement by a factor of 1.5 in the vertical plane by the 2D GRIN lens (Figure 10f).

### 4.2. Microwave NDE

Microwave NDE experiments were performed with the 2D GRIN lens to validate its use as an imaging device. Microwaves have the ability to penetrate deep into low-loss dielectrics and hence are suitable for inspection of electrically insulated low-loss composites. In addition to this, a microwave NDE system offers various advantages over other existing NDE techniques such as non-contact, no requirement for couplants, a relatively low cost and one-sided scanning [57]. Therefore, the capability of the fabricated metasurface lens for the detection of defects with far-field microwave NDE data is demonstrated.

**Figure 11:**
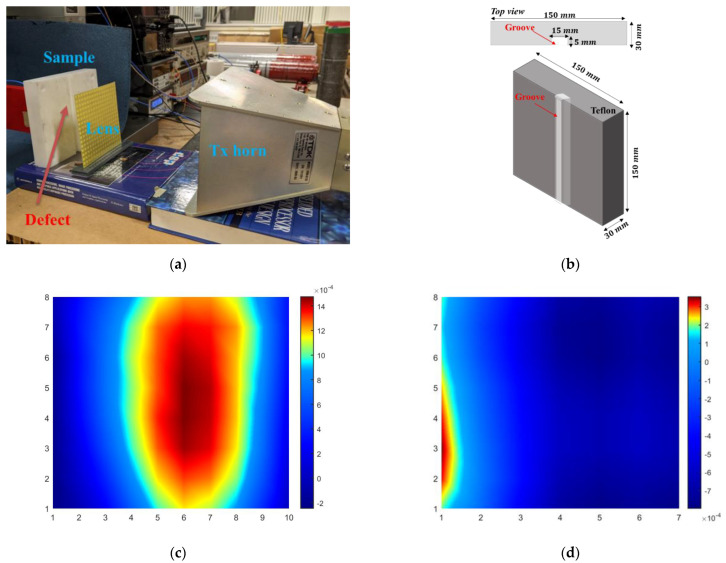
(**a**) Microwave NDE experiment setup. (**b**) Schematic of Teflon sample under test with machined groove. (**c**) Microwave imaging results of groove defect with lens and (**d**) without lens.

The experiment setup is shown in Figure 11a. A machined groove of dimensions 15 mm × 5 mm along the length of the Teflon sample under test was treated as the defect. The contributions due to the defect were measured by subtracting the signal from a similar healthy Teflon sample. The details and flowchart of the defect imaging method are reported in [34]. The sample schematic and details are shown in Figure 11b. The sample was located at the focal plane of the lens. A quarter-wave monopole was used to scan the vertical plane and sample the field distribution in transmission mode. The presence of the groove defect is indicated by the presence of the 1D strip maxima shown in Figure 11c. The experiments were performed without the lens (Figure 11d) to show that the groove defect can only be defected due to the focusing action obtained using the proposed GRIN metasurface lens.

## 5. Conclusions

The emergence of metamaterials and metasurfaces in the past two decades has provided unprecedented capabilities for engineering novel lenses in the microwave regime. Gradient index metasurface lenses offer several advantages over other metamaterial lens designs. This paper presents the design of a GRIN metasurface lens operating at microwave frequencies. Numerical analysis of the metasurface unit cell is described to guide the lens design. A prototype of the proposed lens was fabricated for experimental verification and the focusing action of the lens was studied using field distributions. The numerical and experimental results for focusing are found to be in good agreement, validating the design. The proposed lens uses PCB technology and hence provides a low-cost and low-profile design.

The authors have earlier studied the feasibility of using NIM lenses for microwave non-destructive evaluation (NDE) of glass fiber-reinforced polymer (GFRP) composites. While it was demonstrated that NIM lenses can provide subwavelength focusing and defect detection capabilities, they can only focus waves from a diverging source kept at an appropriate distance from the lens. GRIN metasurface lens designs allow the focusing of plane waves with no restrictions on the source or focal spot distances. A focal spot size of 0.65λ was obtained at a distance of 1.67λ for a NIM lens operating at 6.3 GHz [58] whereas a focal spot size of 1.2λ is achieved with the proposed GRIN lens at a distance of 8.26λ. Moreover, the GRIN lens also provides a planar, low-loss and lightweight design that is suitable for fabrication and integration with microwave imaging systems. Future work will involve the extensive application of the 2D GRIN Metasurface lens for microwave imaging. 

## Data Availability

Not applicable.
